# Supplementary material for: Intestinal microbiology shapes population health impacts of diet and lifestyle risk exposures in Torres Strait Islander communities
Source: eLife. 2020 Oct 19;9:e58407. doi: 10.7554/eLife.58407 (PMC7572126; doi:10.7554/eLife.58407)
Supplement: Supplementary file 1. [file elife-58407-supp1.docx]

**SUPPLEMENTARY FILE 1**

**Intestinal microbiology shapes population health impacts of diet and lifestyle risk exposures in Torres Strait Islander communities:**

***Supplementary Tables***

| **Supplementary File 1a:** Species clusters according to exploratory factor analysis (EFA) with a regression score loading > \|0.1\|. | |
| --- | --- |
| **Species** | **Cluster** |
| Acidaminococcus | 5 |
| Akkermansia muciniphila | 4 |
| Alistipes finegoldii | 6 |
| Alistipes indistinctus | 6 |
| Alistipes onderdonkii | 6 |
| Alistipes putredinis | 6 |
| Alistipes senegalensis | 6 |
| Alistipes shahii | 6 |
| Anaerostipes hadrus | 1 |
| Bacteroidales bacterium ph8 | 6 |
| Bacteroides caccae | 6 |
| Bacteroides cellulosilyticus | 5 |
| Bacteroides dorei | 5 |
| Bacteroides finegoldii | 5 |
| Bacteroides fragilis | 1 |
| Bacteroides massiliensis | 6 |
| Bacteroides ovatus | 1 |
| Bacteroides stercoris | 5 |
| Bacteroides thetaiotaomicron | 5 |
| Bacteroides uniformis | 6 |
| Bacteroides vulgatus | 5 |
| Bacteroides xylanisolvens | 5 |
| Barnesiella intestinihominis | 6 |
| Bifidobacterium adolescentis | 4 |
| Bifidobacterium bifidum | 6 |
| Bifidobacterium catenulatum | 4 |
| Bifidobacterium longum | 3 |
| Bilophila | 6 |
| Bilophila wadsworthia | 6 |
| Burkholderiales bacterium 1 1 47 | 2 |
| Catenibacterium mitsuokai | 4 |
| Clostridiales bacterium 1 7 47FAA | 5 |
| Clostridium asparagiforme | 5 |
| Clostridium bolteae | 5 |
| Clostridium citroniae | 5 |
| Clostridium hathewayi | 4 |
| Collinsella aerofaciens | 4 |
| Coprococcus catus | 3 |
| Coprococcus comes | 6 |
| Dorea formicigenerans | 2 |
| Dorea longicatena | 4 |
| Escherichia coli | 3 |
| Escherichia | 3 |
| Eubacterium biforme | 6 |
| Eubacterium eligens | 3 |
| Eubacterium hallii | 1 |
| Eubacterium ramulus | 2 |
| Eubacterium rectale | 1 |
| Eubacterium siraeum | 4 |
| Eubacterium ventriosum | 6 |
| Faecalibacterium prausnitzii | 1 |
| Flavonifractor plautii | 5 |
| Haemophilus parainfluenzae | 1 |
| Holdemania | 5 |
| Klebsiella pneumoniae | 4 |
| Lachnospiraceae bacterium 3 1 46FAA | 5 |
| Lachnospiraceae bacterium 3 1 57FAA CT1 | 4 |
| Lachnospiraceae bacterium 5 1 63FAA | 1 |
| Lachnospiraceae bacterium 7 1 58FAA | 4 |
| Lachnospiraceae bacterium 8 1 57FAA | 1 |
| Methanobrevibacter smithii | 4 |
| Methanobrevibacter | 4 |
| Odoribacter splanchnicus | 6 |
| Oscillibacter | 5 |
| Parabacteroides distasonis | 5 |
| Parabacteroides merdae | 6 |
| Parabacteroides | 5 |
| Paraprevotella | 6 |
| Parasutterella excrementihominis | 2 |
| Phascolarctobacterium succinatutens | 4 |
| Prevotella copri | 3 |
| Roseburia hominis | 2 |
| Roseburia intestinalis | 1 |
| Roseburia inulinivorans | 2 |
| Rothia mucilaginosa | 1 |
| Ruminococcus bromii | 4 |
| Ruminococcus callidus | 4 |
| Ruminococcus gnavus | 1 |
| Ruminococcus lactaris | 3 |
| Ruminococcus obeum | 1 |
| Ruminococcus torques | 3 |
| Streptococcus parasanguinis | 6 |
| Streptococcus salivarius | 6 |
| Subdoligranulum | 4 |
| Sutterella wadsworthensis | 5 |
| Veillonella atypica | 1 |
| Veillonella parvula | 1 |
| Veillonella | 1 |

| **Supplementary File 1b:** Relative abundance of dominant phyla, by island | | | | |  |
| --- | --- | --- | --- | --- | --- |
|  | **Waiben***^1^* | **Mer***^1^* | **P value***^2^* | **P value***^3^* | |
| **Firmicutes** | 51.00 ± 20.53 | 46.79 ± 16.73 | 0.587 | 0.585 | |
| **Bacteroidetes** | 37.04 ± 19.45 | 36.05 ± 18.94 | 0.697 | 0.697 | |
| **Proteobacteria** | 3.32 ± 6.04 | 5.96 ± 7.99 | **<0.001** | **<0.001** | |
| **Verrucomicrobia** | 2.65 ± 7.88 | 3.78 ± 7.71 | 0.064 | 0.065 | |
| **Actinobacteria** | 5.92 ± 7.96 | 7.19 ± 11.60 | 0.872 | 0.872 | |
| **Euryarchaeota** | 0.05 ± 0.13 | 1.14 ± 0.27 | **0.015** | **0.015** | |
| *^1^ Results are mean ± SD relative abundance. ^2^ P values obtained from ANOVA on log-transformed values. ^3^ P values obtained from age-adjusted ANCOVA on log-transformed values* | | | | | |

| **Supplementary File 1c:** Comparison of the equivalent models of the final structural equation model. | | | | | | | |
| --- | --- | --- | --- | --- | --- | --- | --- |
| **Model** | **DF** | **χ^2^ *P*** | **CFI** | **TLI** | **AIC** | **RMSEA*** | **SRMR** |
| Final model | 102 | 0.058^†^ | 0.926^†^ | 0.707 | 2751^†^ | 0.061^†^ (0.040, 0.078) | 0.044^†^ |
| **Model2** | 107 | 0.048 | 0.859 | 0.670 | 4493 | 0.087 (0.073, 0.101) | 0.048 |
| **Model3** | 107 | 0.010 | 0.762 | 0.565 | 4517 | 0.087 (0.073, 0.101) | 0.048 |
| **Model4** | 110 | <0.001 | 0.737 | 0.529 | 4529 | 0.090 (0.077, 0.104) | 0.067 |
| DF, degrees of freedom; χ^2^ P, Chi-squared p-value; CFI, comparative fix index; TLI, Tucker Lewis Index; AIC, Akaike information criterion; RSMEA, root mean square error of approximation 90%(CI); SRMR, standardized root mean residuals; Confidence Interval; CI. * RMSEA (90%CI). ^†^ Indicates a good model fit: χ^2^ P > 0.05; CFI > 0.8; RMSEA < 0.05, and SRME < 0.08.  Final model: the gut microbiome mediates the exposure-inflammation relationship; Model 2: the gut microbiome influences pathophysiology but is not influenced by risk exposures; Model 3: the gut microbiome is influenced by exposures but does not influence pathophysiology; Model 4: exposure risk factors are associated with inflammatory profile, and inflammatory profile predicts gut microbiome composition | | | | | | | |

| **Supplementary File 1d:** Role of Lachnospiraceae bacterium 8_1_57FAA in mediating the relation of sugar-sweetened beverage intake with mean arterial pressure | | | | | | |
| --- | --- | --- | --- | --- | --- | --- |
|  | **Average Causal Mediation Effect** | | **Average Direct Effect** | | **Total effect** | |
|  | Estimate (95% CI) | **P value** | **Estimate** (95% CI) | **P value** | **Estimate** (95% CI) | **P value** |
|  |  |  |  |  |  |  |
| MEAN ARTERIAL PRESSURE |  |  |  |  |  |  |
| Lachnospiraceae bacterium 8 1 57FAA |  |  |  |  |  |  |
| Unadjusted | 0.691 (0.102, 1.570) | **0.020** | -0.697 (-2.600, 1.020) | 0.410 | -0.006 (-1.530, 1.420) | 0.950 |
| Age-adjusted | 0.706 (0.093, 1.560) | **0.024** | 0.136 (-1.864, 1.980) | 0.898 | 0.842 (-0.822, 2.520) | 0.300 |
| Age- and site-adjusted | 0.726 (0.104, 1.570) | **0.016** | 0.222 (-1.776, 2.160) | 0.860 | 0.948 (-0.694, 2.670) | 0.246 |
| Multivariate-adjusted ^1^ | 0.654 (0.001, 1.660) | **0.054** | -0.359 (-2.591, 1.660) | 0.672 | 0.295 (-1.730, 2.170) | 0.808 |
| *Statistical significance of the average direct, indirect, and total effects was tested using bootstrapping procedures. ^1^ Includes adjustment for age, island, body mass index, gender, cigarette use, and intakes of fruits, vegetables, takeaways, seafood and alcohol.* | | | | | | |

| **Supplementary File 1e:** Key resources table | | |  |  |
| --- | --- | --- | --- | --- |
| **REAGENT TYPE (SPECIES) OR RESOURCE** | **DESIGNATION** | **SOURCE OR REFERENCE** | **IDENTIFIERS** | **ADDITIONAL INFORMATION** |
| **Sample collection kits** | | |  |  |
| commercial assay or kit | OMNIgene GUT OMR-200 | DNAGenotek, Ontario, Canada | N/A |  |
| commercial assay or kit | HS 8-plex Human ProcartaPlex Panel | Thermofisher scientific, MA, USA | catalogue# QGP-308 |  |
| commercial assay or kit | HS 9-plex Human ProcartaPlex Panel | Thermofisher scientific, MA, USA | catalogue# EPXS090-12199-901 |  |
| commercial assay or kit | CRP Human ProcartaPlex Simplex Kit | Thermofisher scientific, MA, USA | RRID:AB_2575812 |  |
| commercial assay or kit | Folate AccuBind ELISA Kit | Monobind Inc, CA, USA | catalogue# 7525-300 |  |
|  | Human LBP ELISA Kit | Hycult Biotech Inc, PA, USA | RRID:AB_10989485 |  |
| **DNA isolation and library prep kits** | | |  |  |
| commercial assay or kit | DNeasy Powerlyzer PowerSoil kit | Qiagen, Hilden, Germany | Cat# 12855-100 |  |
| commercial assay or kit | Qubit Fluorometric Quantification | Thermofisher scientific, MA, USA | catalogue# Q33226 |  |
| commercial assay or kit | Quant-iT HS dsDNA Assay Kit | Thermofisher scientific, MA, USA | catalogue# Q33130 |  |
| commercial assay or kit | Nextera XT Library Preparation Kit v2 | Illumina Inc., CA, USA | Cat# FCF-131-1096 |  |
| commercial assay or kit | Nextera XT Index kit v2 Set A | Illumina Inc., CA, USA | Cat# FC-131-2001 |  |
| **DNA sequencing** |  |  |  |  |
| commercial assay or kit | HiSeq 2500 PE kit v2 | Illumina Inc., CA, USA | Cat# PE-402-4002 |  |
|  | Illumina HiSeq 2500 system | Illumina Inc., CA, USA | N/A |  |
| **Software and algorithms** | | |  |  |
| software, algorithm | bcl2fastq (version 1.8.4) | Illumina Inc., CA, USA |  | <http://sapac.support.illumina.com/downloads/bcl2fastq> |
| software, algorithm | FastQC (version 0.11.5 ) | Babraham Institute |  | https://www.bioinformatics.babraham.ac.uk/projects/fastqc/ |
| software, algorithm | Trimmomatic (version 0.36 ) | Bolger et al. 2014 |  | http://www.usadellab.org/cms/?page=trimmomatic |
| software, algorithm | MetaPhlAn2 (version 2.6.0) | Truong et al., 2015 |  | https://bitbucket.org/biobakery/metaphlan2 |
| software, algorithm | HUMAnN2 (version 0.11.1) | Franzosa et al., 2018 |  | https://bitbucket.org/biobakery/humann2/ |
| software, algorithm | Bowtie2 (version 2.2.4 ) | Langmead & Salzberg 2012 |  | https://sourceforge.net/projects/bowtie-bio/files/bowtie2/2.2.4/ |
| software, algorithm | GraPhlAn (version 0.9.7) | Asnicar et al., 2015 |  | https://bitbucket.org/nsegata/graphlan/ |
| software, algorithm | LEfSe (version 1.0) | Segata et al., 2011 |  | https://bitbucket.org/nsegata/lefse |
| software, algorithm | Primer6 (version 6.1.16) | PRIMER-E, New Zealand |  | https://www.primer-e.com/ |
| software, algorithm | R (version 3.5.2) | R Core Team |  | https://www.r-project.org/ |
| software, algorithm | GraphPad Prism 7 (version 7.04) | GraphPad Software, Inc |  | https://bitbucket.org/nsegata/graphlan/ |
| software, algorithm | Cytoscape (version 3.7.1) | Cytoscape Consortium |  | https://cytoscape.org/ |
| software, algorithm | SAS/STAT software (version 9.4) | SAS Institute |  | https://www.sas.com/en_au/software/stat.html |

| **Supplementary File 1f:** Summary of the quality-controlled faecal microbiome sequencing reads used in this study | | | | | | | |  |  |
| --- | --- | --- | --- | --- | --- | --- | --- | --- | --- |
| **Sequencing ID** | **SRA Accession^†^** | **Study site** | **Length *^a^*** | **Length *^b^*** | **Total sequences *^c^*** | **Adapter content** | **Poor quality reads^*^** |  |  |
| 001_VA | SAMN10383104 | Waiben | 111.16 | 107.54 | 16,314,350 | pass | 0 |  |  |
| 004_RF | SAMN10383105 | Waiben | 110.12 | 106.80 | 18,850,586 | pass | 0 |  |  |
| 011_MA | SAMN10383106 | Waiben | 111.19 | 107.40 | 22,224,208 | pass | 0 |  |  |
| 015_MA | SAMN10383107 | Waiben | 111.17 | 107.93 | 17,569,372 | pass | 0 |  |  |
| 019_LG | SAMN10383108 | Waiben | 110.86 | 108.34 | 21,961,098 | pass | 0 |  |  |
| 020_KN | SAMN10383109 | Waiben | 110.24 | 108.50 | 26,247,172 | pass | 0 |  |  |
| 021_JC | SAMN10383110 | Waiben | 110.16 | 108.00 | 24,464,962 | pass | 0 |  |  |
| 022_SM | SAMN10383111 | Waiben | 110.29 | 107.64 | 22,624,436 | pass | 0 |  |  |
| 024_JM | SAMN10383112 | Waiben | 113.85 | 112.24 | 9,227,046 | pass | 0 |  |  |
| 029_UW | SAMN10383113 | Waiben | 110.32 | 108.79 | 20,919,624 | pass | 0 |  |  |
| 037_ST | SAMN10383114 | Waiben | 110.31 | 107.10 | 15,927,262 | pass | 0 |  |  |
| 040_KB | SAMN10383115 | Waiben | 110.31 | 107.22 | 22,094,734 | pass | 0 |  |  |
| 041_MNL | SAMN10383116 | Waiben | 110.05 | 108.08 | 18,986,830 | pass | 0 |  |  |
| 042_SPA | SAMN10383117 | Waiben | 110.40 | 107.78 | 19,402,432 | pass | 0 |  |  |
| 045_NM | SAMN10383118 | Waiben | 109.97 | 107.60 | 13,918,620 | pass | 0 |  |  |
| 046_RM | SAMN10383119 | Waiben | 110.07 | 107.68 | 20,648,836 | pass | 0 |  |  |
| 052_WC | SAMN10383120 | Waiben | 112.42 | 108.61 | 16,841,710 | pass | 0 |  |  |
| 053_MA | SAMN10383121 | Waiben | 112.22 | 108.49 | 13,678,484 | pass | 0 |  |  |
| 060_DW | SAMN10383122 | Waiben | 112.58 | 109.06 | 16,947,588 | pass | 0 |  |  |
| 061_NA | SAMN10383123 | Waiben | 112.39 | 109.21 | 15,728,302 | pass | 0 |  |  |
| 064_WAW | SAMN10383124 | Waiben | 112.60 | 109.28 | 12,886,736 | pass | 0 |  |  |
| 065_MWA | SAMN10383125 | Waiben | 112.37 | 109.30 | 12,306,184 | pass | 0 |  |  |
| 067_RM | SAMN10383126 | Waiben | 112.61 | 109.02 | 12,875,508 | pass | 0 |  |  |
| 069_EJK | SAMN10383127 | Waiben | 112.35 | 109.74 | 14,048,066 | pass | 0 |  |  |
| 071_KC | SAMN10383128 | Waiben | 112.61 | 109.20 | 12,090,234 | pass | 0 |  |  |
| 074_DC | SAMN10383129 | Waiben | 112.64 | 109.68 | 14,000,902 | pass | 0 |  |  |
| 075_SM | SAMN10383130 | Waiben | 112.46 | 109.36 | 13,156,342 | pass | 0 |  |  |
| 078_AT | SAMN10383131 | Waiben | 110.60 | 107.28 | 22,581,878 | pass | 0 |  |  |
| 079_MC | SAMN10383132 | Waiben | 110.02 | 107.71 | 21,657,480 | pass | 0 |  |  |
| 082_SW | SAMN10383133 | Waiben | 110.30 | 107.68 | 21,420,264 | pass | 0 |  |  |
| 083_AP | SAMN10383134 | Waiben | 110.27 | 108.67 | 25,525,924 | pass | 0 |  |  |
| 084_JP | SAMN10383135 | Waiben | 110.69 | 108.44 | 22,690,840 | pass | 0 |  |  |
| 088_PB | SAMN10383136 | Waiben | 110.40 | 108.40 | 16,742,078 | pass | 0 |  |  |
| 089_AA | SAMN10383137 | Waiben | 110.14 | 108.15 | 24,595,614 | pass | 0 |  |  |
| 091_FC | SAMN10383138 | Waiben | 111.70 | 107.59 | 15,037,926 | pass | 0 |  |  |
| 092_TP | SAMN10383139 | Waiben | 111.34 | 107.90 | 16,377,528 | pass | 0 |  |  |
| 093_SD | SAMN10383140 | Waiben | 111.32 | 107.34 | 17,913,462 | pass | 0 |  |  |
| 094_MG | SAMN10383141 | Waiben | 111.54 | 108.26 | 20,107,958 | pass | 0 |  |  |
| 095_SB | SAMN10383142 | Waiben | 111.81 | 107.98 | 17,243,732 | pass | 0 |  |  |
| 098_LD | SAMN10383143 | Waiben | 111.52 | 107.77 | 16,341,514 | pass | 0 |  |  |
| 100_BC | SAMN10383144 | Waiben | 110.99 | 108.11 | 20,883,598 | pass | 0 |  |  |
| 101_MB | SAMN10383145 | Waiben | 110.41 | 107.60 | 21,884,938 | pass | 0 |  |  |
| 103_ML | SAMN10383146 | Waiben | 110.33 | 107.99 | 20,665,386 | pass | 0 |  |  |
| 104_GL | SAMN10383147 | Waiben | 110.06 | 107.12 | 16,163,006 | pass | 0 |  |  |
| 106_FC | SAMN10383148 | Waiben | 110.47 | 108.28 | 19,503,702 | pass | 0 |  |  |
| 107_ZC | SAMN10383149 | Waiben | 110.29 | 107.55 | 20,137,058 | pass | 0 |  |  |
| 111_AG | SAMN10383150 | Waiben | 110.68 | 108.58 | 21,992,576 | pass | 0 |  |  |
| 113_KD | SAMN10383151 | Waiben | 110.03 | 107.80 | 23,392,176 | pass | 0 |  |  |
| 115_FR | SAMN10383152 | Waiben | 110.51 | 106.58 | 12,461,522 | pass | 0 |  |  |
| 122_KM | SAMN10383153 | Waiben | 110.53 | 107.89 | 15,504,684 | pass | 0 |  |  |
| 302_TK | SAMN10383154 | Mer | 109.93 | 108.32 | 18,841,546 | pass | 0 |  |  |
| 303_EG | SAMN10383155 | Mer | 110.11 | 107.80 | 19,479,558 | pass | 0 |  |  |
| 304_RK | SAMN10383156 | Mer | 110.42 | 107.49 | 16,452,584 | pass | 0 |  |  |
| 305_FK | SAMN10383157 | Mer | 110.38 | 108.20 | 18,747,102 | pass | 0 |  |  |
| 306_RS | SAMN10383158 | Mer | 109.99 | 107.85 | 20,952,788 | pass | 0 |  |  |
| 307_GK | SAMN10383159 | Mer | 110.07 | 107.00 | 14,825,382 | pass | 0 |  |  |
| 308_VM | SAMN10383160 | Mer | 109.47 | 107.11 | 20,041,478 | pass | 0 |  |  |
| 309_PK | SAMN10383161 | Mer | 110.07 | 108.02 | 16,751,954 | pass | 0 |  |  |
| 310_GM | SAMN10383162 | Mer | 110.33 | 107.29 | 15,769,036 | pass | 0 |  |  |
| 311_MK | SAMN10383163 | Mer | 110.25 | 106.93 | 15,953,164 | pass | 0 |  |  |
| 312_KN | SAMN10383164 | Mer | 110.21 | 107.82 | 13,766,938 | pass | 0 |  |  |
| 313_VM | SAMN10383165 | Mer | 109.78 | 107.44 | 16,139,388 | pass | 0 |  |  |
| 314_SP | SAMN10383166 | Mer | 110.20 | 107.80 | 16,325,656 | pass | 0 |  |  |
| 315_SK | SAMN10383167 | Mer | 109.92 | 108.05 | 23,291,160 | pass | 0 |  |  |
| 317_TG | SAMN10383168 | Mer | 110.09 | 107.03 | 15,626,526 | pass | 0 |  |  |
| 319_DM | SAMN10383169 | Mer | 109.99 | 107.66 | 21,248,986 | pass | 0 |  |  |
| 320_ST | SAMN10383170 | Mer | 109.90 | 106.37 | 15,794,838 | pass | 0 |  |  |
| 321_RM | SAMN10383171 | Mer | 109.28 | 105.88 | 17,376,814 | pass | 0 |  |  |
| 322_LT | SAMN10383172 | Mer | 109.99 | 107.44 | 17,388,214 | pass | 0 |  |  |
| 324_AT | SAMN10383173 | Mer | 110.26 | 106.48 | 17,212,410 | pass | 0 |  |  |
| 329_NW | SAMN10383174 | Mer | 110.60 | 107.01 | 20,211,908 | pass | 0 |  |  |
| 332_MW | SAMN10383175 | Mer | 110.12 | 107.12 | 12,118,392 | pass | 0 |  |  |
| 333_MW | SAMN10383176 | Mer | 109.82 | 107.19 | 23,268,760 | pass | 0 |  |  |
| 334_BT | SAMN10383177 | Mer | 112.23 | 108.97 | 15,268,996 | pass | 0 |  |  |
| 335_LK | SAMN10383178 | Mer | 112.30 | 108.57 | 12,722,544 | pass | 0 |  |  |
| 336_PT | SAMN10383179 | Mer | 112.54 | 108.46 | 16,483,958 | pass | 0 |  |  |
| 337_TP | SAMN10383180 | Mer | 112.25 | 108.71 | 12,901,304 | pass | 0 |  |  |
| 338_EC | SAMN10383181 | Mer | 112.21 | 108.39 | 13,428,958 | pass | 0 |  |  |
| 339_GK | SAMN10383182 | Mer | 111.98 | 107.36 | 11,364,864 | pass | 0 |  |  |
| 340_CM | SAMN10383183 | Mer | 111.61 | 108.03 | 16,034,732 | pass | 0 |  |  |
| 341_ST | SAMN10383184 | Mer | 112.08 | 108.03 | 11,146,812 | pass | 0 |  |  |
| 343_VB | SAMN10383185 | Mer | 112.29 | 108.00 | 13,592,686 | pass | 0 |  |  |
| 344_SB | SAMN10383186 | Mer | 112.14 | 108.25 | 10,370,806 | pass | 0 |  |  |
| 345_MK | SAMN10383187 | Mer | 112.19 | 108.54 | 12,780,066 | pass | 0 |  |  |
| 346_JS | SAMN10383188 | Mer | 112.14 | 109.26 | 18,546,208 | pass | 0 |  |  |
| 347_RT | SAMN10383189 | Mer | 112.56 | 109.35 | 11,701,068 | pass | 0 |  |  |
| 348_DO | SAMN10383190 | Mer | 112.49 | 109.22 | 11,366,288 | pass | 0 |  |  |
| 349_GS | SAMN10383191 | Mer | 112.80 | 108.54 | 8,829,566 | pass | 0 |  |  |
| 352_AB | SAMN10383192 | Mer | 112.44 | 109.32 | 11,101,176 | pass | 0 |  |  |
| 354_AK | SAMN10383193 | Mer | 112.68 | 109.51 | 12,457,090 | pass | 0 |  |  |
| 357_WT | SAMN10383194 | Mer | 112.63 | 108.57 | 7,381,250 | pass | 0 |  |  |
| 359_AB | SAMN10383195 | Mer | 111.70 | 108.20 | 11,046,358 | pass | 0 |  |  |
| 360_JG | SAMN10383196 | Mer | 112.72 | 108.92 | 9,344,478 | pass | 0 |  |  |
| 364_JZ | SAMN10383197 | Mer | 112.87 | 108.75 | 13,926,718 | pass | 0 |  |  |
| 367_RL | SAMN10383198 | Mer | 112.66 | 108.61 | 9,269,766 | pass | 0 |  |  |
| 370_WH | SAMN10383199 | Mer | 112.60 | 109.09 | 10,092,762 | pass | 0 |  |  |
| 371_SK | SAMN10383200 | Mer | 112.51 | 108.78 | 10,543,158 | pass | 0 |  |  |
| 379_JP | SAMN10383201 | Mer | 110.43 | 108.21 | 24,755,278 | pass | 0 |  |  |
| 382_JK | SAMN10383202 | Mer | 110.50 | 108.74 | 19,777,296 | pass | 0 |  |  |
| 385_CP | SAMN10383203 | Mer | 110.58 | 107.89 | 18,305,744 | pass | 0 |  |  |
| *^a^ Average length of forward reads; ^b^ Average length of reverse reads; ^c^ Total number of paired-end reads post-QC; ^*^ Number of sequences flagged as poor quality using FastQC; ^†^ Sequence Read Archive (SRA) bio-sample accession number in bio-project number PRJNA503909.* | | | | | | | |  |  |

**References**

Asnicar F, Weingart G, Tickle TL, Huttenhower C, Segata N. 2015. Compact graphical representation of phylogenetic data and metadata with GraPhlAn. PeerJ 3:e1029. DOI: <https://doi.org/10.7717/peerj.1029>, PMID: 26157614

Bolger AM, Lohse M, Usadel B. 2014. Trimmomatic: a flexible trimmer for illumina sequence data. Bioinformatics 30:2114–2120. DOI: <https://doi.org/10.1093/bioinformatics/btu170>, PMID: 24695404

Franzosa EA, McIver LJ, Rahnavard G, Thompson LR, Schirmer M, Weingart G, Lipson KS, Knight R, Caporaso JG, Segata N, Huttenhower C. 2018. Species-level functional profiling of metagenomes and metatranscriptomes. Nature Methods 15:962–968. DOI: <https://doi.org/10.1038/s41592-018-0176-y>, PMID: 30377376

Langmead B and Salzberg SL. 2012. Fast gapped-read alignment with Bowtie 2. Nature methods, 9(4), p.357. DOI: <https://doi.org/10.1038/nmeth.1923>, PMID: 22388286

R Development Core Team. 2010. R: a language and environment for statistical computing. 3.2.2 . Vienna, Austria, R Foundation for Statistical Computing. <https://www.R-project.org/>

Segata N, Izard J, Waldron L, Gevers D, Miropolsky L, Garrett WS, Huttenhower C. 2011. Metagenomic biomarker discovery and explanation. Genome Biology, 12:R60. DOI: <https://doi.org/10.1186/gb-2011-12-6-r60>, PMID: 21702898
